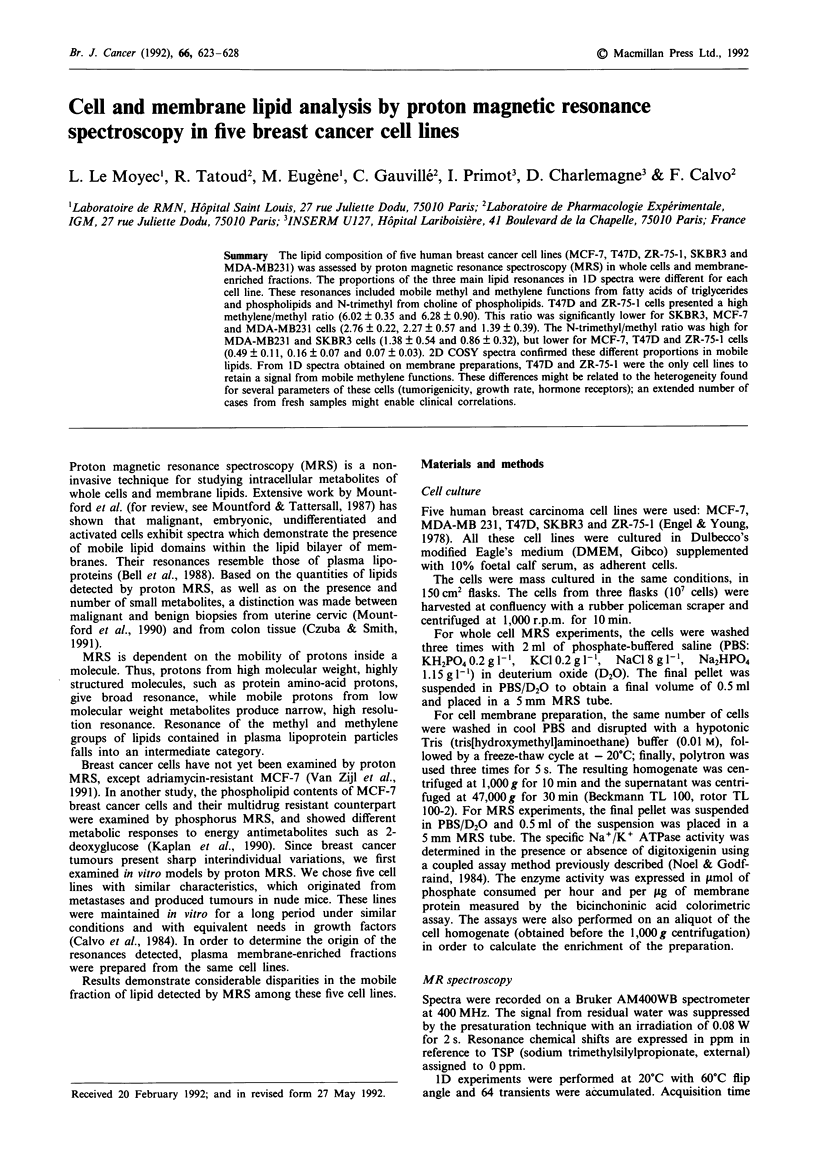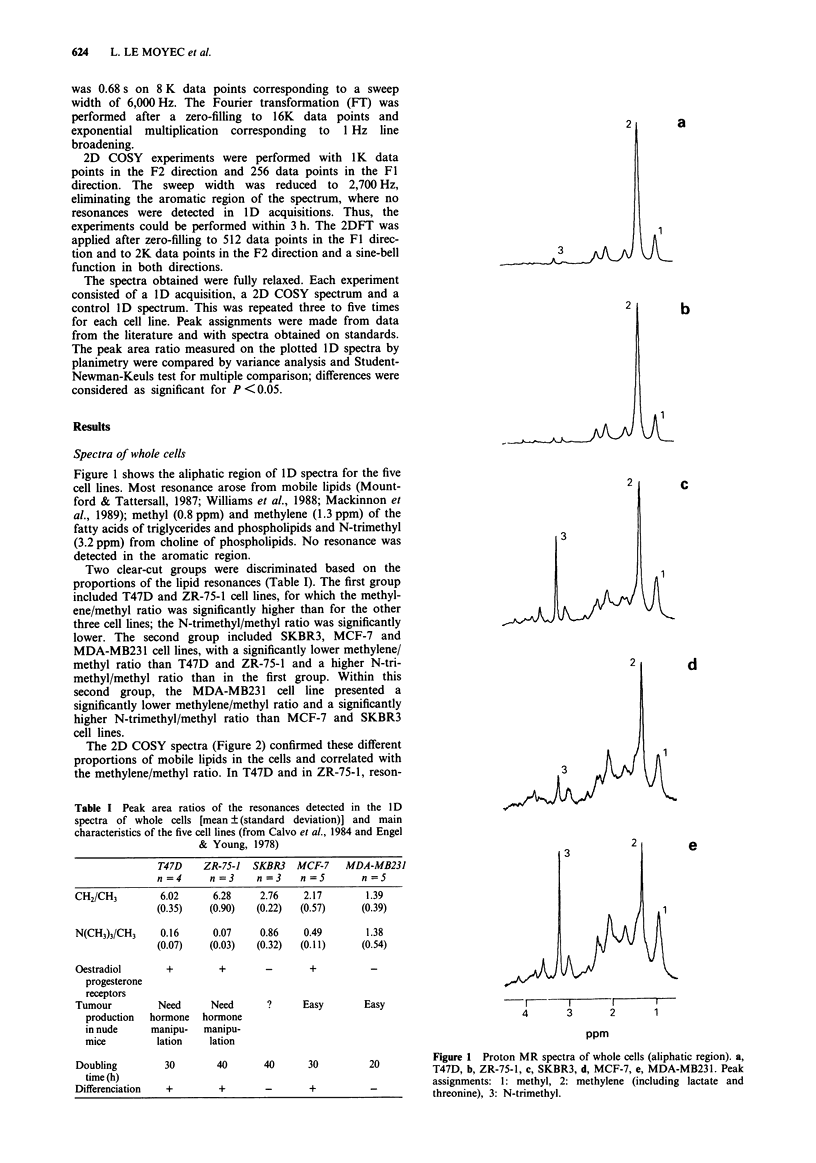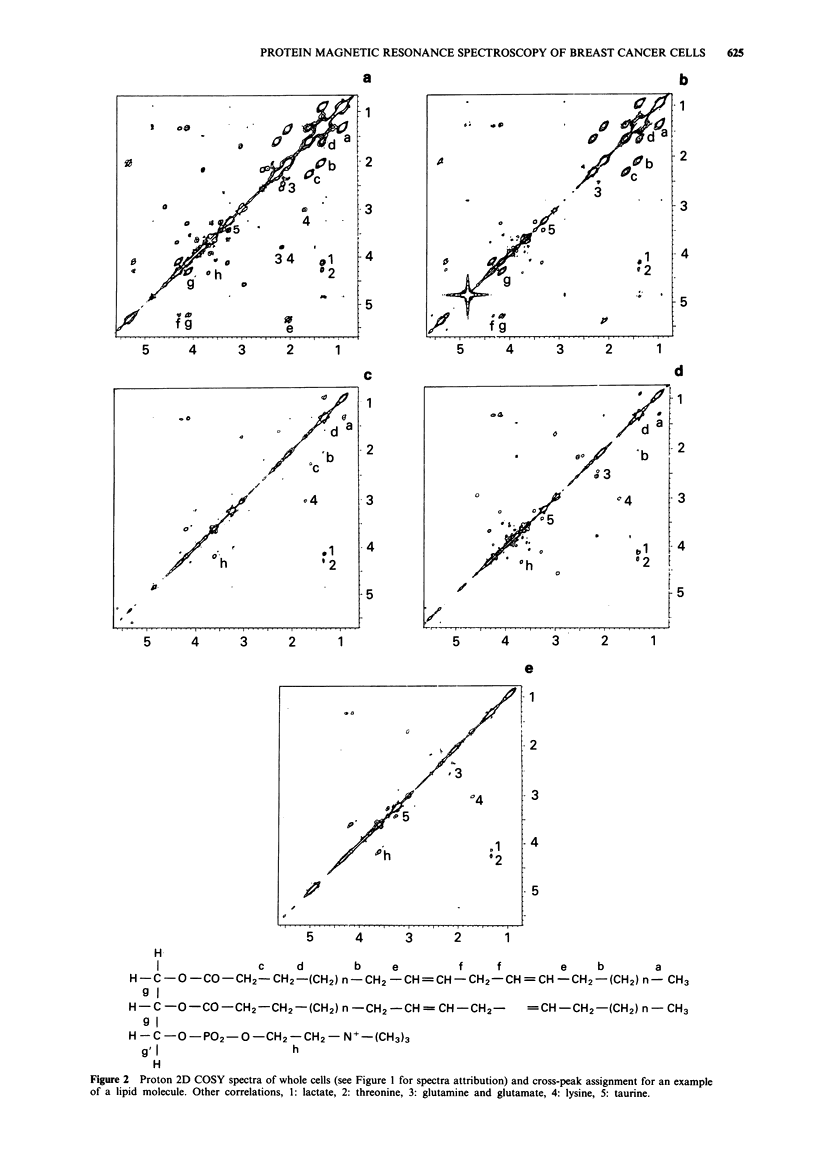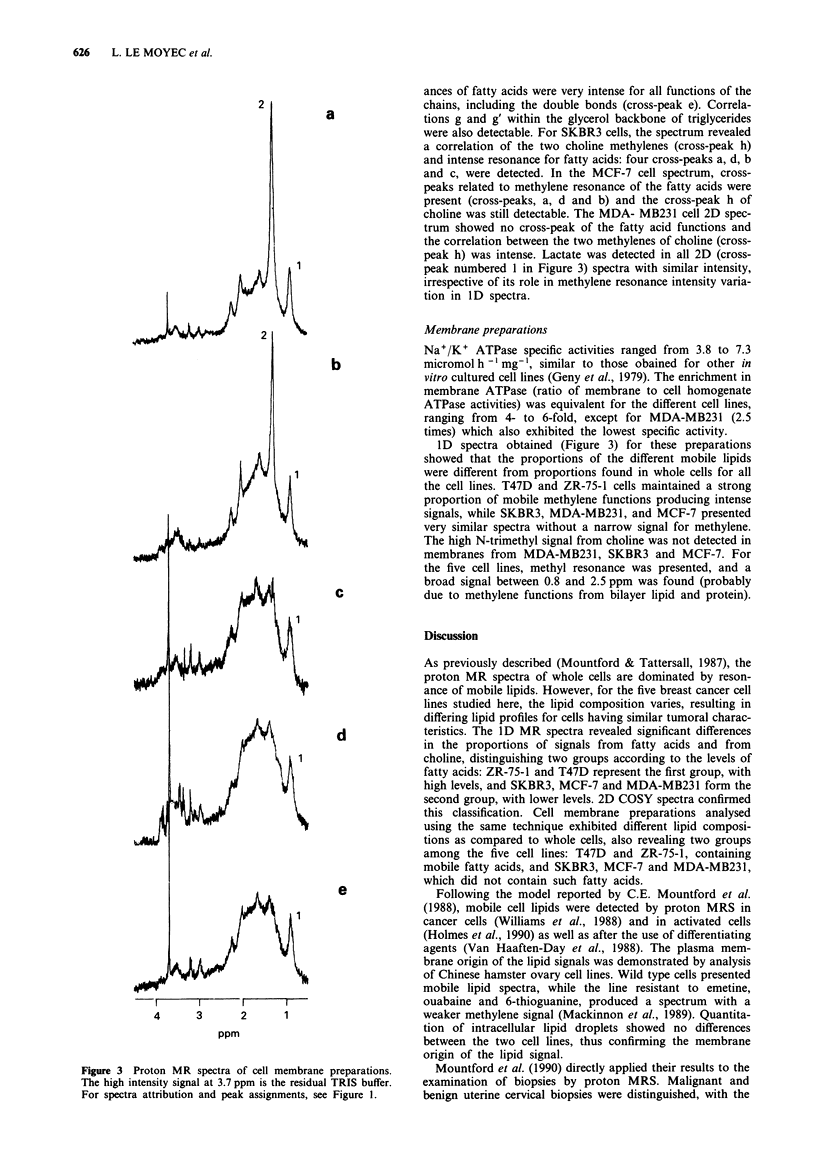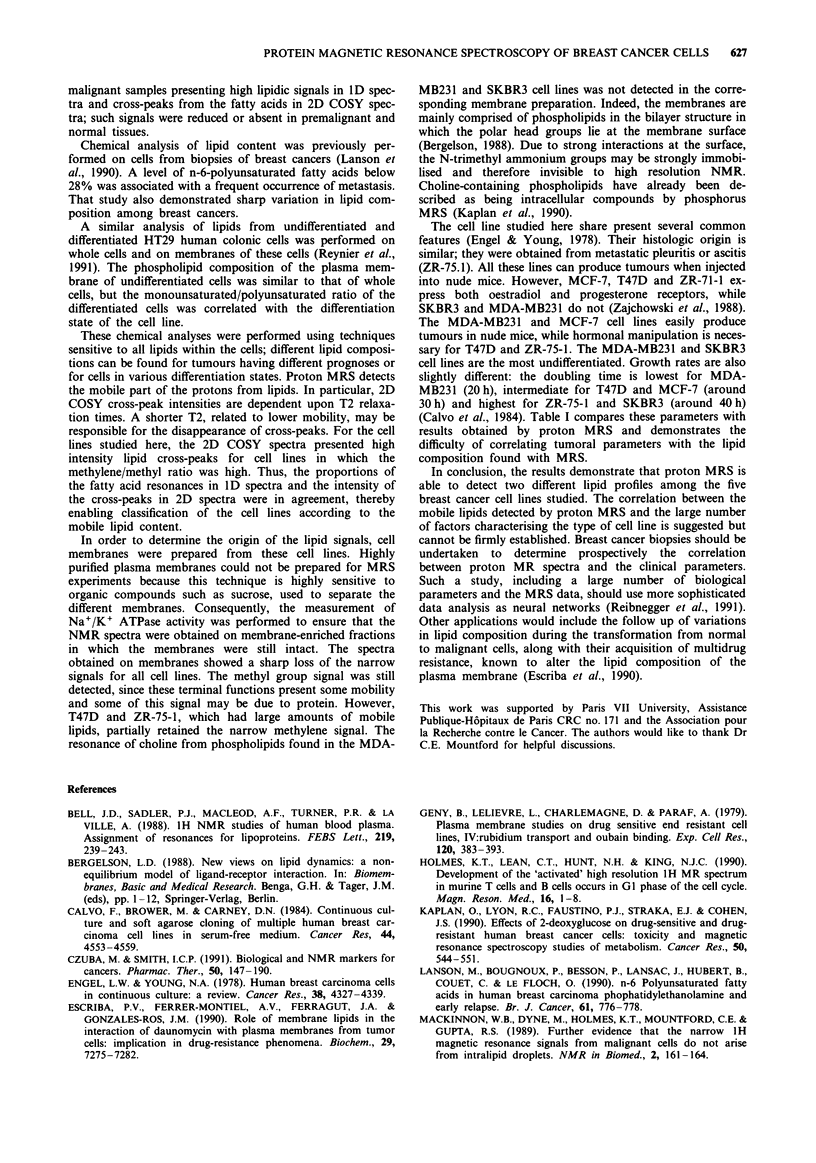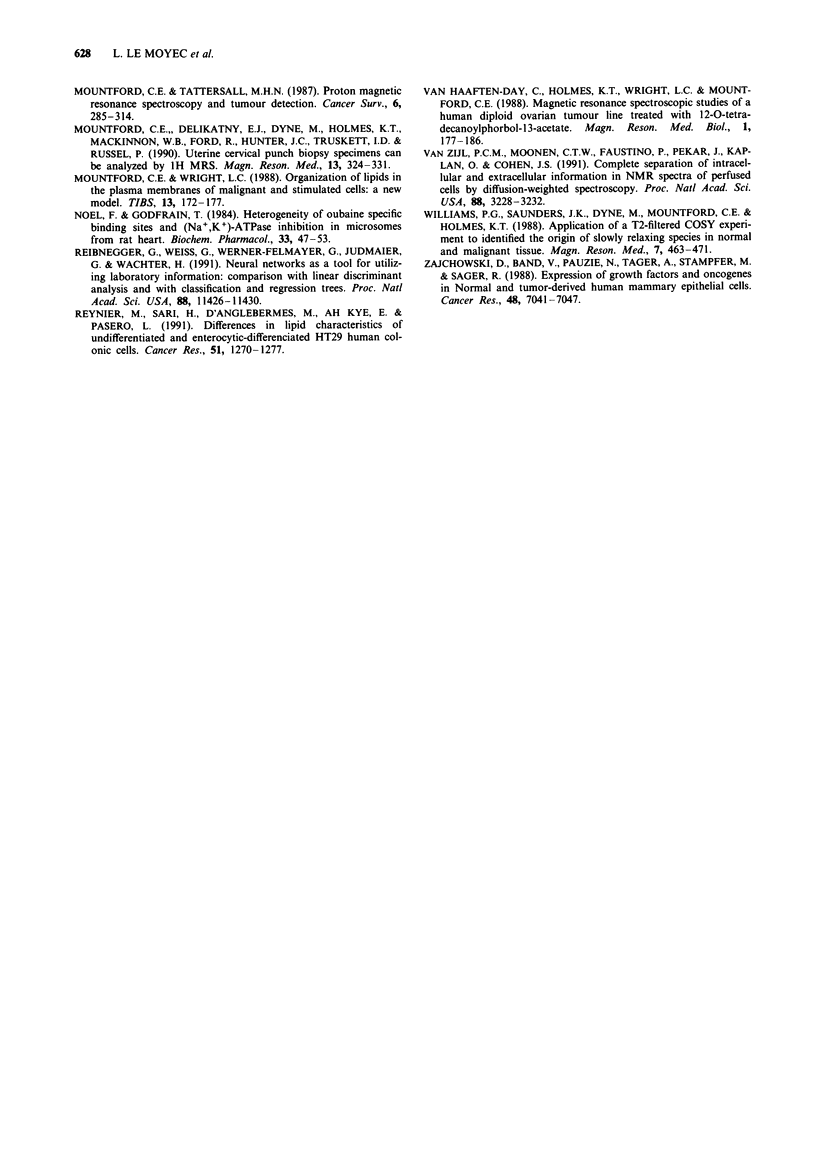# Cell and membrane lipid analysis by proton magnetic resonance spectroscopy in five breast cancer cell lines.

**DOI:** 10.1038/bjc.1992.327

**Published:** 1992-10

**Authors:** L. Le Moyec, R. Tatoud, M. Eugène, C. Gauvillé, I. Primot, D. Charlemagne, F. Calvo

**Affiliations:** Laboratoire de RMN, Hôpital Saint Louis, Paris, France.

## Abstract

The lipid composition of five human breast cancer cell lines (MCF-7, T47D, ZR-75-1, SKBR3 and MDA-MB231) was assessed by proton magnetic resonance spectroscopy (MRS) in whole cells and membrane-enriched fractions. The proportions of the three main lipid resonances in 1D spectra were different for each cell line. These resonances included mobile methyl and methylene functions from fatty acids of triglycerides and phospholipids and N-trimethyl from choline of phospholipids. T47D and ZR-75-1 cells presented a high methylene/methyl ratio (6.02 +/- 0.35 and 6.28 +/- 0.90). This ratio was significantly lower for SKBR3, MCF-7 and MDA-MB231 cells (2.76 +/- 0.22, 2.27 +/- 0.57 and 1.39 +/- 0.39). The N-trimethyl/methyl ratio was high for MDA-MB231 and SKBR3 cells (1.38 +/- 0.54 and 0.86 +/- 0.32), but lower for MCF-7, T47D and ZR-75-1 cells (0.49 +/- 0.11, 0.16 +/- 0.07 and 0.07 +/- 0.03). 2D COSY spectra confirmed these different proportions in mobile lipids. From 1D spectra obtained on membrane preparations, T47D and ZR-75-1 were the only cell lines to retain a signal from mobile methylene functions. These differences might be related to the heterogeneity found for several parameters of these cells (tumorigenicity, growth rate, hormone receptors); an extended number of cases from fresh samples might enable clinical correlations.


Br. J. Cancer (1992), 66, 623-628 © Macmillan Press Ltd., 1992

### Cell and membrane lipid analysis by proton magnetic resonance spectroscopy in five breast cancer cell lines

L. Le Moyec[1], R. Tatoud[2], M. Eugène[1], C. Gauvillé[2], I. Primot[3], D. Charlemagne[3] & F. Calvo[2]

[1]*Laboratoire de RMN, Hôpital Saint Louis, 27 rue Juliette Dodu, 75010 Paris;* [2]*Laboratoire de Pharmacologie Expérimentale, IGM, 27 rue Juliette Dodu, 75010 Paris;* [3]*INSERM U127, Hôpital Lariboisière, 41 Boulevard de la Chapelle, 75010 Paris; France*

**Summary** The lipid composition of five human breast cancer cell lines (MCF-7, T47D, ZR-75-1, SKBR3 and MDA-MB231) was assessed by proton magnetic resonance spectroscopy (MRS) in whole cells and membrane-enriched fractions. The proportions of the three main lipid resonances in 1D spectra were different for each cell line. These resonances included mobile methyl and methylene functions from fatty acids of triglycerides and phospholipids and N-trimethyl from choline of phospholipids. T47D and ZR-75-1 cells presented a high methylene/methyl ratio ($6.02 \pm 0.35$ and $6.28 \pm 0.90$). This ratio was significantly lower for SKBR3, MCF-7 and MDA-MB231 cells ($2.76 \pm 0.22$, $2.27 \pm 0.57$ and $1.39 \pm 0.39$). The N-trimethyl/methyl ratio was high for MDA-MB231 and SKBR3 cells ($1.38 \pm 0.54$ and $0.86 \pm 0.32$), but lower for MCF-7, T47D and ZR-75-1 cells ($0.49 \pm 0.11$, $0.16 \pm 0.07$ and $0.07 \pm 0.03$). 2D COSY spectra confirmed these different proportions in mobile lipids. From 1D spectra obtained on membrane preparations, T47D and ZR-75-1 were the only cell lines to retain a signal from mobile methylene functions. These differences might be related to the heterogeneity found for several parameters of these cells (tumorigenicity, growth rate, hormone receptors); an extended number of cases from fresh samples might enable clinical correlations.

Proton magnetic resonance spectroscopy (MRS) is a non-invasive technique for studying intracellular metabolites of whole cells and membrane lipids. Extensive work by Mountford *et al.* (for review, see Mountford & Tattersall, 1987) has shown that malignant, embryonic, undifferentiated and activated cells exhibit spectra which demonstrate the presence of mobile lipid domains within the lipid bilayer of membranes. Their resonances resemble those of plasma lipoproteins (Bell *et al.*, 1988). Based on the quantities of lipids detected by proton MRS, as well as on the presence and number of small metabolites, a distinction was made between malignant and benign biopsies from uterine cervic (Mountford *et al.*, 1990) and from colon tissue (Czuba & Smith, 1991).

MRS is dependent on the mobility of protons inside a molecule. Thus, protons from high molecular weight, highly structured molecules, such as protein amino-acid protons, give broad resonance, while mobile protons from low molecular weight metabolites produce narrow, high resolution resonance. Resonance of the methyl and methylene groups of lipids contained in plasma lipoprotein particles falls into an intermediate category.

Breast cancer cells have not yet been examined by proton MRS, except adriamycin-resistant MCF-7 (Van Zijl *et al.*, 1991). In another study, the phospholipid contents of MCF-7 breast cancer cells and their multidrug resistant counterpart were examined by phosphorus MRS, and showed different metabolic responses to energy antimetabolites such as 2-deoxyglucose (Kaplan *et al.*, 1990). Since breast cancer tumours present sharp interindividual variations, we first examined *in vitro* models by proton MRS. We chose five cell lines with similar characteristics, which originated from metastases and produced tumours in nude mice. These lines were maintained *in vitro* for a long period under similar conditions and with equivalent needs in growth factors (Calvo *et al.*, 1984). In order to determine the origin of the resonances detected, plasma membrane-enriched fractions were prepared from the same cell lines.

Results demonstrate considerable disparities in the mobile fraction of lipid detected by MRS among these five cell lines.

Received 20 February 1992; and in revised form 27 May 1992.

### Materials and methods

### *Cell culture*

Five human breast carcinoma cell lines were used: MCF-7, MDA-MB 231, T47D, SKBR3 and ZR-75-1 (Engel & Young, 1978). All these cell lines were cultured in Dulbecco's modified Eagle's medium (DMEM, Gibco) supplemented with 10% foetal calf serum, as adherent cells.

The cells were mass cultured in the same conditions, in 150 $cm^2$ flasks. The cells from three flasks ($10^7$ cells) were harvested at confluency with a rubber policeman scraper and centrifuged at 1,000 r.p.m. for 10 min.

For whole cell MRS experiments, the cells were washed three times with 2 ml of phosphate-buffered saline (PBS: $KH_2PO_4$ 0.2 g $l^{-1}$, KCl 0.2 g $l^{-1}$, NaCl 8 g $l^{-1}$, $Na_2HPO_4$ 1.15 g $l^{-1}$) in deuterium oxide ($D_2O$). The final pellet was suspended in PBS/$D_2O$ to obtain a final volume of 0.5 ml and placed in a 5 mm MRS tube.

For cell membrane preparation, the same number of cells were washed in cool PBS and disrupted with a hypotonic Tris (tris[hydroxymethyl]aminoethane) buffer (0.01 M), followed by a freeze-thaw cycle at − 20°C; finally, polytron was used three times for 5 s. The resulting homogenate was centrifuged at 1,000 *g* for 10 min and the supernatant was centrifuged at 47,000 *g* for 30 min (Beckmann TL 100, rotor TL 100-2). For MRS experiments, the final pellet was suspended in PBS/$D_2O$ and 0.5 ml of the suspension was placed in a 5 mm MRS tube. The specific $Na^+/K^+$ ATPase activity was determined in the presence or absence of digitoxigenin using a coupled assay method previously described (Noel & Godfraind, 1984). The enzyme activity was expressed in μmol of phosphate consumed per hour and per μg of membrane protein measured by the bicinchoninic acid colorimetric assay. The assays were also performed on an aliquot of the cell homogenate (obtained before the 1,000 *g* centrifugation) in order to calculate the enrichment of the preparation.

### *MR spectroscopy*

Spectra were recorded on a Bruker AM400WB spectrometer at 400 MHz. The signal from residual water was suppressed by the presaturation technique with an irradiation of 0.08 W for 2 s. Resonance chemical shifts are expressed in ppm in reference to TSP (sodium trimethylsilylpropionate, external) assigned to 0 ppm.

1D experiments were performed at 20°C with 60°C flip angle and 64 transients were accumulated. Acquisition time was 0.68 s on 8 K data points corresponding to a sweep width of 6,000 Hz. The Fourier transformation (FT) was performed after a zero-filling to 16K data points and exponential multiplication corresponding to 1 Hz line broadening.

2D COSY experiments were performed with 1K data points in the F2 direction and 256 data points in the F1 direction. The sweep width was reduced to 2,700 Hz, eliminating the aromatic region of the spectrum, where no resonances were detected in 1D acquisitions. Thus, the experiments could be performed within 3 h. The 2DFT was applied after zero-filling to 512 data points in the F1 direction and to 2K data points in the F2 direction and a sine-bell function in both directions.

The spectra obtained were fully relaxed. Each experiment consisted of a 1D acquisition, a 2D COSY spectrum and a control 1D spectrum. This was repeated three to five times for each cell line. Peak assignments were made from data from the literature and with spectra obtained on standards. The peak area ratio measured on the plotted 1D spectra by planimetry were compared by variance analysis and Student-Newman-Keuls test for multiple comparison; differences were considered as significant for $P < 0.05$.

### Results

### *Spectra of whole cells*

Figure 1 shows the aliphatic region of 1D spectra for the five cell lines. Most resonance arose from mobile lipids (Mountford & Tattersall, 1987; Williams *et al.*, 1988; Mackinnon *et al.*, 1989); methyl (0.8 ppm) and methylene (1.3 ppm) of the fatty acids of triglycerides and phospholipids and N-trimethyl (3.2 ppm) from choline of phospholipids. No resonance was detected in the aromatic region.

Two clear-cut groups were discriminated based on the proportions of the lipid resonances (Table I). The first group included T47D and ZR-75-1 cell lines, for which the methylene/methyl ratio was significantly higher than for the other three cell lines; the N-trimethyl/methyl ratio was significantly lower. The second group included SKBR3, MCF-7 and MDA-MB231 cell lines, with a significantly lower methylene/methyl ratio than T47D and ZR-75-1 and a higher N-trimethyl/methyl ratio than in the first group. Within this second group, the MDA-MB231 cell line presented a significantly lower methylene/methyl ratio and a significantly higher N-trimethyl/methyl ratio than MCF-7 and SKBR3 cell lines.

The 2D COSY spectra (Figure 2) confirmed these different proportions of mobile lipids in the cells and correlated with the methylene/methyl ratio. In T47D and in ZR-75-1, reson-

**Table I** Peak area ratios of the resonances detected in the 1D spectra of whole cells [mean ± (standard deviation)] and main characteristics of the five cell lines (from Calvo *et al.*, 1984 and Engel & Young, 1978)

| | *T47D* $n = 4$ | *ZR-75-1* $n = 3$ | *SKBR3* $n = 3$ | *MCF-7* $n = 5$ | *MDA-MB231* $n = 5$ |
|---|---|---|---|---|---|
| $CH_2/CH_3$ | 6.02 (0.35) | 6.28 (0.90) | 2.76 (0.22) | 2.17 (0.57) | 1.39 (0.39) |
| $N(CH_3)_3/CH_3$ | 0.16 (0.07) | 0.07 (0.03) | 0.86 (0.32) | 0.49 (0.11) | 1.38 (0.54) |
| Oestradiol progesterone receptors | + | + | – | + | – |
| Tumour production in nude mice | Need hormone manipulation | Need hormone manipulation | ? | Easy | Easy |
| Doubling time (h) | 30 | 40 | 40 | 30 | 20 |
| Differenciation | + | + | – | + | – |

**Figure 1** Proton MR spectra of whole cells (aliphatic region). **a**, T47D, **b**, ZR-75-1, **c**, SKBR3, **d**, MCF-7, **e**, MDA-MB231. Peak assignments: 1: methyl, 2: methylene (including lactate and threonine), 3: N-trimethyl.

**Figure 2** Proton 2D COSY spectra of whole cells (see Figure 1 for spectra attribution) and cross-peak assignment for an example of a lipid molecule. Other correlations, 1: lactate, 2: threonine, 3: glutamine and glutamate, 4: lysine, 5: taurine.

**Figure 3** Proton MR spectra of cell membrane preparations. The high intensity signal at 3.7 ppm is the residual TRIS buffer. For spectra attribution and peak assignments, see Figure 1.

ances of fatty acids were very intense for all functions of the chains, including the double bonds (cross-peak e). Correlations g and g′ within the glycerol backbone of triglycerides were also detectable. For SKBR3 cells, the spectrum revealed a correlation of the two choline methylenes (cross-peak h) and intense resonance for fatty acids: four cross-peaks a, d, b and c, were detected. In the MCF-7 cell spectrum, cross-peaks related to methylene resonance of the fatty acids were present (cross-peaks, a, d and b) and the cross-peak h of choline was still detectable. The MDA- MB231 cell 2D spectrum showed no cross-peak of the fatty acid functions and the correlation between the two methylenes of choline (cross-peak h) was intense. Lactate was detected in all 2D (cross-peak numbered 1 in Figure 3) spectra with similar intensity, irrespective of its role in methylene resonance intensity variation in 1D spectra.

*Membrane preparations*

$Na^+/K^+$ ATPase specific activities ranged from 3.8 to 7.3 micromol $h^{-1}$ $mg^{-1}$, similar to those obained for other *in vitro* cultured cell lines (Geny *et al.*, 1979). The enrichment in membrane ATPase (ratio of membrane to cell homogenate ATPase activities) was equivalent for the different cell lines, ranging from 4- to 6-fold, except for MDA-MB231 (2.5 times) which also exhibited the lowest specific activity.

1D spectra obtained (Figure 3) for these preparations showed that the proportions of the different mobile lipids were different from proportions found in whole cells for all the cell lines. T47D and ZR-75-1 cells maintained a strong proportion of mobile methylene functions producing intense signals, while SKBR3, MDA-MB231, and MCF-7 presented very similar spectra without a narrow signal for methylene. The high N-trimethyl signal from choline was not detected in membranes from MDA-MB231, SKBR3 and MCF-7. For the five cell lines, methyl resonance was presented, and a broad signal between 0.8 and 2.5 ppm was found (probably due to methylene functions from bilayer lipid and protein).

### Discussion

As previously described (Mountford & Tattersall, 1987), the proton MR spectra of whole cells are dominated by resonance of mobile lipids. However, for the five breast cancer cell lines studied here, the lipid composition varies, resulting in differing lipid profiles for cells having similar tumoral characteristics. The 1D MR spectra revealed significant differences in the proportions of signals from fatty acids and from choline, distinguishing two groups according to the levels of fatty acids: ZR-75-1 and T47D represent the first group, with high levels, and SKBR3, MCF-7 and MDA-MB231 form the second group, with lower levels. 2D COSY spectra confirmed this classification. Cell membrane preparations analysed using the same technique exhibited different lipid compositions as compared to whole cells, also revealing two groups among the five cell lines: T47D and ZR-75-1, containing mobile fatty acids, and SKBR3, MCF-7 and MDA-MB231, which did not contain such fatty acids.

Following the model reported by C.E. Mountford *et al.* (1988), mobile cell lipids were detected by proton MRS in cancer cells (Williams *et al.*, 1988) and in activated cells (Holmes *et al.*, 1990) as well as after the use of differentiating agents (Van Haaften-Day *et al.*, 1988). The plasma membrane origin of the lipid signals was demonstrated by analysis of Chinese hamster ovary cell lines. Wild type cells presented mobile lipid spectra, while the line resistant to emetine, ouabaine and 6-thioguanine, produced a spectrum with a weaker methylene signal (Mackinnon *et al.*, 1989). Quantitation of intracellular lipid droplets showed no differences between the two cell lines, thus confirming the membrane origin of the lipid signal.

Mountford *et al.* (1990) directly applied their results to the examination of biopsies by proton MRS. Malignant and benign uterine cervical biopsies were distinguished, with the malignant samples presenting high lipidic signals in 1D spectra and cross-peaks from the fatty acids in 2D COSY spectra; such signals were reduced or absent in premalignant and normal tissues.

Chemical analysis of lipid content was previously performed on cells from biopsies of breast cancers (Lanson *et al.*, 1990). A level of n-6-polyunsaturated fatty acids below 28% was associated with a frequent occurrence of metastasis. That study also demonstrated sharp variation in lipid composition among breast cancers.

A similar analysis of lipids from undifferentiated and differentiated HT29 human colonic cells was performed on whole cells and on membranes of these cells (Reynier *et al.*, 1991). The phospholipid composition of the plasma membrane of undifferentiated cells was similar to that of whole cells, but the monounsaturated/polyunsaturated ratio of the differentiated cells was correlated with the differentiation state of the cell line.

These chemical analyses were performed using techniques sensitive to all lipids within the cells; different lipid compositions can be found for tumours having different prognoses or for cells in various differentiation states. Proton MRS detects the mobile part of the protons from lipids. In particular, 2D COSY cross-peak intensities are dependent upon T2 relaxation times. A shorter T2, related to lower mobility, may be responsible for the disappearance of cross-peaks. For the cell lines studied here, the 2D COSY spectra presented high intensity lipid cross-peaks for cell lines in which the methylene/methyl ratio was high. Thus, the proportions of the fatty acid resonances in 1D spectra and the intensity of the cross-peaks in 2D spectra were in agreement, thereby enabling classification of the cell lines according to the mobile lipid content.

In order to determine the origin of the lipid signals, cell membranes were prepared from these cell lines. Highly purified plasma membranes could not be prepared for MRS experiments because this technique is highly sensitive to organic compounds such as sucrose, used to separate the different membranes. Consequently, the measurement of $Na^+/K^+$ ATPase activity was performed to ensure that the NMR spectra were obtained on membrane-enriched fractions in which the membranes were still intact. The spectra obtained on membranes showed a sharp loss of the narrow signals for all cell lines. The methyl group signal was still detected, since these terminal functions present some mobility and some of this signal may be due to protein. However, T47D and ZR-75-1, which had large amounts of mobile lipids, partially retained the narrow methylene signal. The resonance of choline from phospholipids found in the MDA-MB231 and SKBR3 cell lines was not detected in the corresponding membrane preparation. Indeed, the membranes are mainly comprised of phospholipids in the bilayer structure in which the polar head groups lie at the membrane surface (Bergelson, 1988). Due to strong interactions at the surface, the N-trimethyl ammonium groups may be strongly immobilised and therefore invisible to high resolution NMR. Choline-containing phospholipids have already been described as being intracellular compounds by phosphorus MRS (Kaplan *et al.*, 1990).

The cell line studied here share present several common features (Engel & Young, 1978). Their histologic origin is similar; they were obtained from metastatic pleuritis or ascitis (ZR-75.1). All these lines can produce tumours when injected into nude mice. However, MCF-7, T47D and ZR-71-1 express both oestradiol and progesterone receptors, while SKBR3 and MDA-MB231 do not (Zajchowski *et al.*, 1988). The MDA-MB231 and MCF-7 cell lines easily produce tumours in nude mice, while hormonal manipulation is necessary for T47D and ZR-75-1. The MDA-MB231 and SKBR3 cell lines are the most undifferentiated. Growth rates are also slightly different: the doubling time is lowest for MDA-MB231 (20 h), intermediate for T47D and MCF-7 (around 30 h) and highest for ZR-75-1 and SKBR3 (around 40 h) (Calvo *et al.*, 1984). Table I compares these parameters with results obtained by proton MRS and demonstrates the difficulty of correlating tumoral parameters with the lipid composition found with MRS.

In conclusion, the results demonstrate that proton MRS is able to detect two different lipid profiles among the five breast cancer cell lines studied. The correlation between the mobile lipids detected by proton MRS and the large number of factors characterising the type of cell line is suggested but cannot be firmly established. Breast cancer biopsies should be undertaken to determine prospectively the correlation between proton MR spectra and the clinical parameters. Such a study, including a large number of biological parameters and the MRS data, should use more sophisticated data analysis as neural networks (Reibnegger *et al.*, 1991). Other applications would include the follow up of variations in lipid composition during the transformation from normal to malignant cells, along with their acquisition of multidrug resistance, known to alter the lipid composition of the plasma membrane (Escriba *et al.*, 1990).

This work was supported by Paris VII University, Assistance Publique-Hôpitaux de Paris CRC no. 171 and the Association pour la Recherche contre le Cancer. The authors would like to thank Dr C.E. Mountford for helpful discussions.

### References

BELL, J.D., SADLER, P.J., MACLEOD, A.F., TURNER, P.R. & LA VILLE, A. (1988). 1H NMR studies of human blood plasma. Assignment of resonances for lipoproteins. *FEBS Lett.*, **219**, 239–243.

BERGELSON, L.D. (1988). New views on lipid dynamics: a non-equilibrium model of ligand-receptor interaction. In: *Biomembranes, Basic and Medical Research*. Benga, G.H. & Tager, J.M. (eds), pp. 1–12, Springer-Verlag, Berlin.

CALVO, F., BROWER, M. & CARNEY, D.N. (1984). Continuous culture and soft agarose cloning of multiple human breast carcinoma cell lines in serum-free medium. *Cancer Res*, **44**, 4553–4559.

CZUBA, M. & SMITH, I.C.P. (1991). Biological and NMR markers for cancers. *Pharmac. Ther.*, **50**, 147–190.

ENGEL, L.W. & YOUNG, N.A. (1978). Human breast carcinoma cells in continuous culture: a review. *Cancer Res.*, **38**, 4327–4339.

ESCRIBA, P.V., FERRER-MONTIEL, A.V., FERRAGUT, J.A. & GONZALES-ROS, J.M. (1990). Role of membrane lipids in the interaction of daunomycin with plasma membranes from tumor cells: implication in drug-resistance phenomena. *Biochem.*, **29**, 7275–7282.

GENY, B., LELIEVRE, L., CHARLEMAGNE, D. & PARAF, A. (1979). Plasma membrane studies on drug sensitive end resistant cell lines, IV:rubidium transport and oubain binding. *Exp. Cell Res.*, **120**, 383–393.

HOLMES, K.T., LEAN, C.T., HUNT, N.H. & KING, N.J.C. (1990). Development of the 'activated' high resolution 1H MR spectrum in murine T cells and B cells occurs in G1 phase of the cell cycle. *Magn. Reson. Med.*, **16**, 1–8.

KAPLAN, O., LYON, R.C., FAUSTINO, P.J., STRAKA, E.J. & COHEN, J.S. (1990). Effects of 2-deoxyglucose on drug-sensitive and drug-resistant human breast cancer cells: toxicity and magnetic resonance spectroscopy studies of metabolism. *Cancer Res.*, **50**, 544–551.

LANSON, M., BOUGNOUX, P., BESSON, P., LANSAC, J., HUBERT, B., COUET, C. & LE FLOCH, O. (1990). n-6 Polyunsaturated fatty acids in human breast carcinoma phophatidylethanolamine and early relapse. *Br. J. Cancer*, **61**, 776–778.

MACKINNON, W.B., DYNE, M., HOLMES, K.T., MOUNTFORD, C.E. & GUPTA, R.S. (1989). Further evidence that the narrow 1H magnetic resonance signals from malignant cells do not arise from intralipid droplets. *NMR in Biomed.*, **2**, 161–164.

MOUNTFORD, C.E. & TATTERSALL, M.H.N. (1987). Proton magnetic resonance spectroscopy and tumour detection. *Cancer Surv.*, **6**, 285–314.

MOUNTFORD, C.E.,, DELIKATNY, E.J., DYNE, M., HOLMES, K.T., MACKINNON, W.B., FORD, R., HUNTER, J.C., TRUSKETT, I.D. & RUSSEL, P. (1990). Uterine cervical punch biopsy specimens can be analyzed by 1H MRS. *Magn. Reson. Med.*, **13**, 324–331.

MOUNTFORD, C.E. & WRIGHT, L.C. (1988). Organization of lipids in the plasma membranes of malignant and stimulated cells: a new model. *TIBS*, **13**, 172–177.

NOEL, F. & GODFRAIN, T. (1984). Heterogeneity of oubaine specific binding sites and $(Na^+,K^+)$-ATPase inhibition in microsomes from rat heart. *Biochem. Pharmacol.*, **33**, 47–53.

REIBNEGGER, G., WEISS, G., WERNER-FELMAYER, G., JUDMAIER, G. & WACHTER, H. (1991). Neural networks as a tool for utilizing laboratory information: comparison with linear discriminant analysis and with classification and regression trees. *Proc. Natl Acad. Sci. USA*, **88**, 11426–11430.

REYNIER, M., SARI, H., D'ANGLEBERMES, M., AH KYE, E. & PASERO, L. (1991). Differences in lipid characteristics of undifferentiated and enterocytic-differenciated HT29 human colonic cells. *Cancer Res.*, **51**, 1270–1277.

VAN HAAFTEN-DAY, C., HOLMES, K.T., WRIGHT, L.C. & MOUNTFORD, C.E. (1988). Magnetic resonance spectroscopic studies of a human diploid ovarian tumour line treated with 12-O-tetradecanoylphorbol-13-acetate. *Magn. Reson. Med. Biol.*, **1**, 177–186.

VAN ZIJL, P.C.M., MOONEN, C.T.W., FAUSTINO, P., PEKAR, J., KAPLAN, O. & COHEN, J.S. (1991). Complete separation of intracellular and extracellular information in NMR spectra of perfused cells by diffusion-weighted spectroscopy. *Proc. Natl Acad. Sci. USA*, **88**, 3228–3232.

WILLIAMS, P.G., SAUNDERS, J.K., DYNE, M., MOUNTFORD, C.E. & HOLMES, K.T. (1988). Application of a T2-filtered COSY experiment to identified the origin of slowly relaxing species in normal and malignant tissue. *Magn. Reson. Med.*, **7**, 463–471.

ZAJCHOWSKI, D., BAND, V., PAUZIE, N., TAGER, A., STAMPFER, M. & SAGER, R. (1988). Expression of growth factors and oncogenes in Normal and tumor-derived human mammary epithelial cells. *Cancer Res.*, **48**, 7041–7047.